# The cost-effectiveness of syphilis screening in pregnant women: a systematic literature review

**DOI:** 10.3389/fpubh.2024.1268653

**Published:** 2024-03-21

**Authors:** Meng Zhang, Hongyan Zhang, Xiaoqing Hui, Huimin Qu, Junfen Xia, Feng Xu, Cannan Shi, Junjian He, Yuan Cao, Mengcai Hu

**Affiliations:** Department of Health Care, The Third Affiliated Hospital of Zhengzhou University, Zhengzhou, Henan, China

**Keywords:** syphilis, screening, cost-effectiveness, pregnant women, review

## Abstract

**Introduction:**

The cost-effectiveness study of syphilis screening in pregnant women has not been synthesized. This study aimed to synthesize the economic evidence on the cost-effectiveness of syphilis screening in pregnant women that might contribute to making recommendations on the future direction of syphilis screening approaches.

**Methods:**

We systematically searched MEDLINE, PubMed, and Web of Science databases for relevant studies published before 19 January 2023 and identified the cost-effectiveness analyses for syphilis screening in pregnant women. The methodological design quality was appraised by the Consolidated Health Economic Evaluation Reporting Standards (CHEERS) 2022 checklist.

**Results:**

In total, 17 literature met the eligibility criteria for a full review. Of the 17 studies, four evaluated interventions using different screening methods, seven assessed a combination of syphilis testing and treatment interventions, three focused on repeat screening intervention, and four evaluated the interventions that integrated syphilis and HIV testing. The most cost-effective strategy appeared to be rapid syphilis testing with high treatment rates in pregnant women who were positive.

**Discussion:**

The cost-effectiveness of syphilis screening for pregnancy has been widely demonstrated. It is very essential to improve the compliance with maternal screening and the treatment rates for positive pregnant women while implementing screening.

## Introduction

Syphilis is caused by the *Treponema pallidum* bacterium, which can be transmitted vertically from mother to child during pregnancy. In 2016, an estimated 98,800 cases of maternal syphilis infections occurred worldwide (1). The rate of syphilis among reproductive-age women increased, leading to an increase in the rate of congenital syphilis (CS) (2). More than half of the pregnancies among women with active syphilis result in adverse pregnancy outcomes that can be avoided (3). Historical data show that untreated syphilis during pregnancy can lead to 25% late abortion or stillbirth, 13% premature or low birthweight, 11% neonatal death, and 20% classic symptoms and signs of syphilis-infected infants (4–6). To achieve the goal of eliminating CS, the World Health Organization regards the provision of syphilis screening and treatment in antenatal care (ANC) services as one of several prevention strategies (7).

The incidence rate of CS seems to be decreasing in several countries with the approaches to prevent syphilis advocated by the WHO (8, 9). Although syphilis is preventable and treatable, the incidence of CS is on a steady rise in high-income countries such as Australia and the United States (10, 11). Mother-to-child transmission (MTCT) of syphilis can occur at any time during gestation if syphilitic pregnant women cannot be identified and treated. In addition to syphilis testing in early pregnancy, high-risk pregnant women also need to be tested again at 28 weeks of gestation and delivery (12).

The prevalence of syphilis has been proven to be closely associated with HIV epidemiologically (13). This may be because the two infections have a common route of transmission, and syphilitic genital ulcer increases the probability of HIV infection (14). However, the coverage for HIV screening is several times higher than for syphilis, and newborns who do not acquire HIV infection still have a high risk of syphilis infection (15, 16). HIV antenatal screening integrated with syphilis testing can not only improve the maternal syphilis screening rate but also prove to be cost-effective due to the inexpensive penicillin treatment (17–19).

Several studies have indicated that the screening and treatment of maternal syphilis as a public health intervention reduces adverse pregnancy and birth outcomes and medical costs and improves the quality of life of pregnant women and newborns (20–22). Health policymakers should make rational use of resources based on different incidence rates and social and economic development in different regions. The economic value obtained from the integration of cost and clinical effect is one of the most important outcomes in the evaluation of the comparative effects of medical interventions. Although the high cost-effectiveness of the screening approach depends on the high incidence rate generally, the cost-effectiveness of maternal screening has been proven in areas with low syphilis incidence (18).

Evidence-based estimates of the cost-effectiveness of screening pregnant women for syphilis help make the case for rational allocation of health resources, improve the efficiency of intervention approaches, and make progress toward elimination. Previous estimates have assessed the cost-effectiveness of screening pregnant women for syphilis based on the local syphilis prevalence, screening strategies, and economic levels. We performed a systematic review of the published evidence on the cost-effectiveness of syphilis screening in pregnant women with respect to the extent to which the evidence on the cost-effectiveness of screening has changed. In addition to this, we aimed to estimate the optimal screening strategies and extract information from cost-effectiveness analyses across different studies to provide a scientific basis for the formulation of a suitable syphilis screening strategy.

## Methods

### Search strategy

We searched MEDLINE, PubMed, and Web of Science for the cost-effectiveness of syphilis antenatal screening literature from the earliest available data in each database to 19 January 2023. We adopted the Preferred Reporting Items for Systematic Review and Meta-analysis (PRISMA) statement for this systematic review (23). To identify relevant economic assessments, we used the following terms: syphilis, *treponema pallidum*, pregnancy, antenatal, vertical transmission, mother-to-child transmission, screening, test, diagnose, cost-effectiveness, economic, cost-utility, and cost–benefit (Table 1). The search strategy was composed of medical subject title (MeSH) terms, free text terms, and AND/OR terms. Duplicates were excluded using the software EndNote X9.

**Table 1:** Proposed keywords for the literature search.

Themes	Proposed keywords
Screening	Screening OR Test OR diagnosis OR diagnose OR screening
Syphilis	Syphilis OR treponema pallidum OR syphilis infected women
Economic evaluations	Cost OR economic OR cost-effectiveness OR cost–benefit OR cost-utility OR ecnomic evaluation
Vertical transmission	Pregnancy OR pregnant OR vertical transmission OR mother-to-child transmission OR antenatal OR perinatal

### Inclusion and exclusion criteria

We selected eligible studies using the following reported inclusion criteria: cost-effectiveness studies based on clinical trials or model-based assessments related to syphilis antenatal screening, papers evaluating syphilis testing approach economic value, and papers written in English or Chinese.

The economic assessments included in this review were cost-effectiveness, cost-utility, or cost–benefit analyses. The outcomes of these studies would include at least one of the following: cost per diagnosis of syphilis in pregnant women, cost per averted adverse birth outcomes, cost per quality-adjusted life years (QALY), and the total cost of screening pregnant women for syphilis approaches. We excluded the studies that focused solely on cost analyses or exclusively assessed the cost-effectiveness of syphilis treatment.

### Data extraction and quality assessment

Two independent reviewers summarized the retrieved results and extracted the information of interest. The differences between the two were discussed and resolved. Data extracted included analysis type, country, target population, sample size, intervention, incidence, perspective, outcomes, and costs.

The economic assessments of the methodological quality of syphilis screening in pregnant women were evaluated by the 28-item Consolidated Health Economic Evaluation Reporting Standards (CHEERS) 2022 checklist (24). For the list, each item was scored using “Yes” if the article met the quality criterion, “Not applicable” if the item does not apply to a particular economic evaluation, and “Not reported” if the information is otherwise not reported.

## Results

### Search results

**Figure 1:**
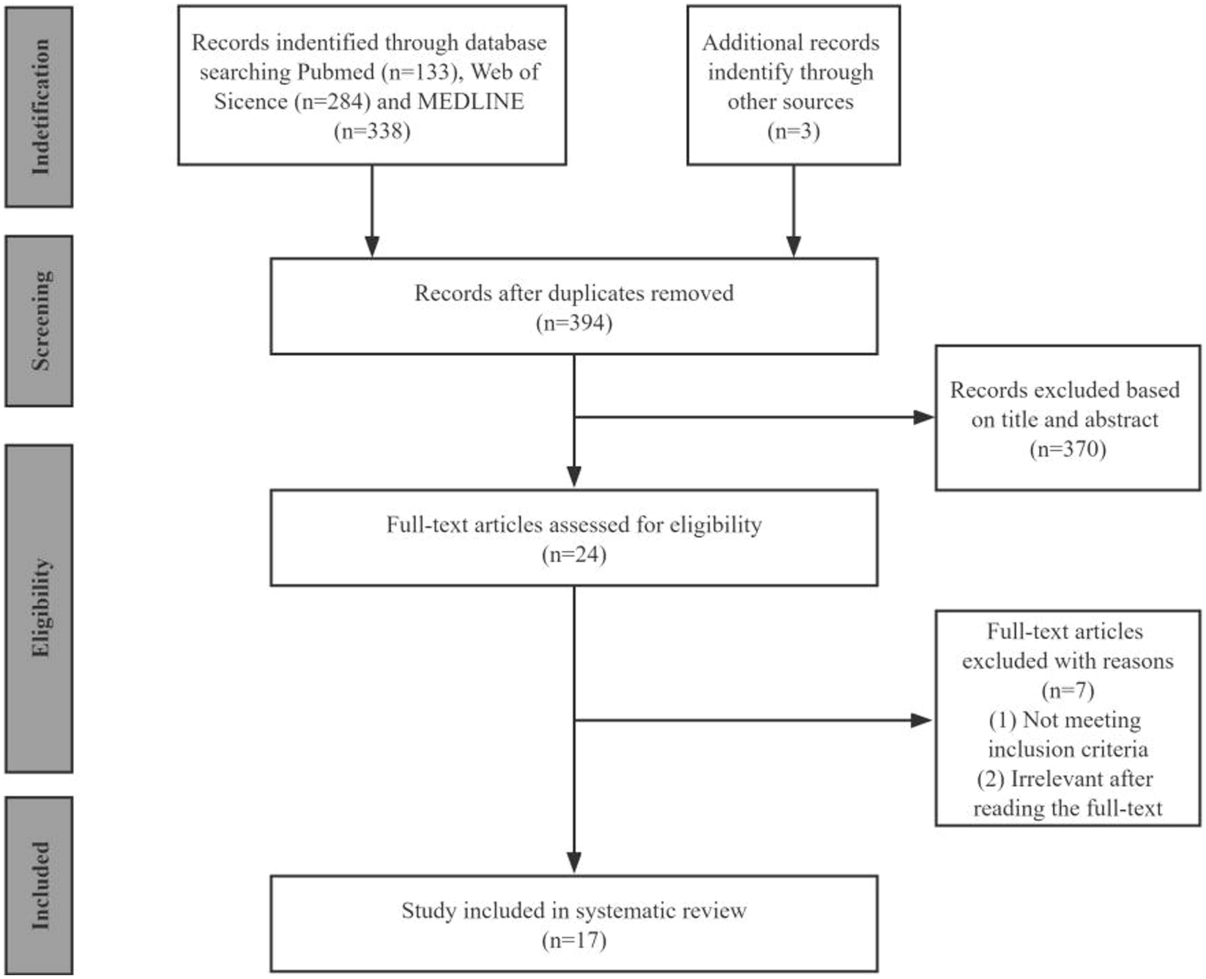
The flow chat of search strategy in a systematic review.

By searching the three databases, we identified 758 publications (PubMed: 133, Web of Science: 284, and MEDLINE: 338). After removing duplicates and excluding irrelevance, we collected 24 articles for full-text review. We identified 17 eligible studies for a final full review as shown in the PRISMA flow diagram (Figure 1).

### Quality assessment

The quality assessment of the 17 studies using the CHEERS 2022 checklist is presented in Table 2. Most of the articles met most of the items in the CHEERS 2022 checklist. The abstracts of most of the articles did not report enough information about the context of highlights and alternative analyses. In contrast to CHEERS 2013, none of the studies met the items related to patient or service recipient, general public, and community or stakeholder involvement and engagement that were added to the CHEERS 2022 checklist. Multiple studies have failed to report where publicly available models can be found, which was encouraged in CHEERS 2022.

**Table 2:** The quality assessment of the 17 studies using the CHEERS 2022 checklist.

	Title	Abstract	Background and objectives	Health economic analysis plan	Study population	Setting and location	Comparators	Perspective	Time horizon	Discount rate	Selection of outcomes	Measurement of outcomes	Valuation of outcomes	Measurement and valuation of resources and costs	Currency, price date, and conversion	Rationale and description of model	Analytics and assumptions	Characterizing heterogeneity	Characterizing distributional effects	Characterizing uncertainty	Approach to engagement with patients and others affected by the study	Study parameters	Summary of the main results	Effect of uncertainty	Effect of engagement with patients and others affected by the study	Study findings, limitations, generalisability, and current knowledge	Source of funding	Conflicts of interest
Vickerman et al. (25)	Y	N	Y	Y	Y	N	Y	N	N	N	Y	Y	Y	Y	Y	Y	Y	N	N	Y	N	Y	Y	Y	N	Y	N	Y
Blandford et al. (26)	Y	N	Y	Y	Y	Y	Y	Y	Y	NA	Y	Y	Y	Y	Y	Y	Y	N	N	N	N	Y	Y	Y	N	Y	N	N
Schackman et al. (27)	Y	N	Y	Y	Y	Y	Y	Y	Y	Y	Y	Y	Y	Y	Y	Y	Y	N	N	Y	N	Y	Y	Y	N	Y	Y	Y
Rydzak and Goldie (28)	Y	N	Y	Y	Y	N	Y	N	Y	N	Y	Y	Y	Y	Y	Y	Y	N	N	Y	N	Y	Y	Y	N	Y	N	N
Owusu-Edusei 2011 (29)	Y	N	Y	Y	Y	Y	Y	N	Y	Y	Y	Y	Y	Y	Y	Y	Y	N	Y	Y	N	Y	Y	Y	N	Y	N	N
Kuznik 2013 (30)	Y	N	Y	Y	Y	Y	Y	Y	Y	Y	Y	Y	Y	Y	Y	Y	Y	Y	Y	Y	N	Y	Y	Y	N	Y	Y	Y
Kahn 2014 (31)	Y	N	Y	Y	Y	N	Y	Y	Y	Y	Y	Y	Y	Y	Y	N	Y	N	Y	Y	N	Y	Y	Y	N	Y	Y	Y
Larson 2014 (22)	Y	N	Y	Y	Y	Y	Y	Y	Y	Y	Y	Y	Y	Y	Y	N	Y	N	N	Y	N	Y	Y	Y	N	Y	Y	Y
Owusu-Edusei et al. (19)	Y	N	Y	Y	Y	Y	Y	Y	Y	Y	Y	Y	Y	Y	Y	Y	Y	N	Y	Y	N	Y	Y	Y	N	Y	NA	Y
Albright et al. (32)	Y	N	Y	Y	Y	Y	Y	Y	Y	Y	Y	Y	Y	Y	Y	Y	Y	N	Y	Y	N	Y	Y	Y	N	Y	NA	Y
Kuznik et al. (33)	Y	N	Y	Y	Y	Y	Y	Y	N	Y	Y	Y	Y	Y	Y	N	Y	Y	Y	Y	N	Y	Y	Y	N	Y	Y	Y
Terris-Prestholt et al. (21)	Y	N	Y	Y	Y	Y	Y	N	N	Y	Y	Y	Y	Y	Y	Y	Y	Y	Y	Y	N	Y	Y	Y	N	Y	N	Y
Bristow et al. (17)	Y	N	Y	Y	Y	Y	Y	Y	Y	Y	Y	Y	Y	Y	Y	Y	Y	N	Y	Y	N	Y	Y	Y	N	Y	Y	Y
Hersh et al. (34)	Y	N	Y	Y	Y	Y	Y	Y	Y	Y	Y	Y	Y	Y	Y	Y	Y	N	Y	Y	N	Y	Y	Y	N	Y	NA	Y
Huntington et al. (35)	Y	Y	Y	Y	Y	Y	Y	Y	Y	Y	Y	Y	Y	Y	Y	Y	Y	N	Y	Y	N	Y	Y	Y	N	Y	NA	Y
Romero et al. (36)	Y	N	Y	Y	Y	Y	Y	Y	Y	Y	Y	Y	Y	Y	Y	Y	Y	N	Y	Y	N	Y	Y	Y	N	Y	Y	Y
Rodriguez et al. (18)	Y	Y	Y	Y	Y	Y	Y	Y	Y	Y	Y	Y	Y	Y	Y	Y	Y	Y	Y	Y	N	Y	Y	Y	N	Y	Y	Y

Y, The paper met the quality criterion; NA, The item does not apply to a particular economic evaluation; and N, Information is otherwise not reported.

### General and economic features of the studies

An overview of the main study characteristics of the 17 included cost-effectiveness studies is shown in Table 3. With the exception of one article that did not identify a specific target setting, the 16 included studies were based on a total of 40 countries or regions. The majority (*N* = 15) of the 16 included studies were based on low-income (*N* = 9) and middle-income (*N* = 6) settings, with the exception of two high-income countries. More than half of the studies were conducted in Africa (*N* = 9), six in America, two in Asia, and two in Europe. The lowest prevalence rate of syphilis among the 17 studies was 0.00419%, and the highest was 10%. In addition to one study targeting 15-year-old girls, 16 studies targeted pregnant women, 12 of which did not identify the characteristics of pregnant women, while two studies identified the age of pregnant women (26 and 10–49 years old, respectively), and two studies identified the gestational weeks of pregnant women (24 and 28–32 weeks, respectively). A majority of the studies (*N* = 13, 76%) were cost-utility analyses (CUAs); of which, outcome measures used disability-adjusted life years (DALYs) (*N* = 12) or quality-adjusted life years (QALYs) (*N* = 1). Four studies performed cost-effectiveness analysis using incremental cost per case averted as an outcome. Of the 17 studies, four studies evaluated interventions using different screening methods, including rapid plasma reagin (RPR), rapid syphilis test, immunochromatographic strip test (ICS), and treponema pallidum hemagglutination assay (TPHA); seven studies assessed the combination of syphilis test and treatment interventions; and three studies focused on repeat screening intervention. In addition, four studies mixed interventions to integrate syphilis and HIV testing.

**Table 3:** The main study characteristics of the 17 included cost-effectiveness studies.

	Country	Intervention	Target population	Syphilis prevalence	Outcomes
Vickerman et al. (25)	Tanzania	I. Syphilis RPR screening. Rapid POC syphilis tests.	Pregnant women	7.2%	Adverting adverse birth outcomes and per DALY saved cost
Blandford et al. (26)	Republic of South Africa	Off-site RPR and all positive samples using the TPHA test and positive patients receiving treatment when returning. On-site RPR test and positive patients receiving treatment on the same day. III. On-site ICS test and positive patients receiving treatment on the same day.	Pregnant women	6.3%	Adverting adverse birth outcomes and incremental cost per case averted
Schackman et al. (27)	Haiti	According to genital ulcer disease found in the prenatal examination, RPR tests, and subsequent treatment performed. An RPR test providing results at the follow-up about 1 week later. A rapid test providing results immediately.	Pregnant women at 24 weeks of gestation	3.8% in rural settings 3.5% in urban settings	ICER
Rydzak and Goldie (28)	Sub-Saharan Africa	No screening. RPR test with TPHA confirmatory test. Single rapid RPR test. Single rapid ICS test.	Girls at 15 years of age	6.0%	Adverting adverse birth outcomes, life expectancy, lifetime costs, and ICER.
Owusu-Ediusei et al. (29)	Sub-Saharan Africa	No screening. Dual-POC test. The RPR and TPHA tests based on laboratories. An on-site RPR test. POC treponemal ICS test.	Pregnant women	10%	Adverting adverse birth outcomes, total cost, DALYs lost
Kuznik et al. (30)	Sub-Saharan Africa	No testing and no treatment. ICS testing and subsequent treatment.	Pregnant women	0.6–14.0%	Adverting adverse birth outcomes and DALYs averted.
Kahn et al. (31)		Scaled-up screening and treatment. Current testing and treatment in ANC.	Pregnant women	0.5–3.0%	Net costs, DALYs averted, and net costs per DALY averted
Larson et al. (22)	Zambia	62% of pregnant women were tested, and 10.4% of positive cases were treated. 62% of pregnant women were tested, and 100% of positive cases were treated. 100% of pregnant women were tested, and 10.4% of positive cases were treated.	Pregnant women	0.83%	Adverting adverse birth outcomes and DALYs
Owusu-Edusei et al. (19)	China	No screening. Single HIV screening and positive patients receiving treatment. Single syphilis screening and positive patients receiving treatment. HIV and syphilis screening and positive for either of the infection and receiving treatment accordingly.	Pregnant women aged 26 years	0.25%	Adverting adverse birth outcomes, total cost, DALYs, CER, and ICER
Albright et al. (32)	the United States	No repeat screening, Repeat screening for syphilis in all women with negative early pregnancy screening.	Pregnant women between 28 and 32 weeks of gestation	0.012%	The cost of adverting adverse birth outcomes and the amount required to repeat screening to prevent one adverse outcome
Kuznik et al. (33)	Low-and Middle-Income Countries in Asia and Latin America	A POC ICS syphilis test and treatment. No testing and no treatment.	Pregnant women	0.1–1.2% in Asia, 0.1–3.9% in Latin America	Adverting adverse birth outcomes and ICER
Terris-Prestholt et al. (21)	Peru Tanzania Zambia	No screening. RPR screening and treatment. Single RST test and treatment. Dual RST++ and treatment. Dual RST+ and treatment. Single RST and treatment by 1st dose, RPR test, and treatment by 2nd and 3rd dose. RPR screening and positive test by Single RST and treatment. Single RST screening and positive test by Dual Trep/Non-Trep RST and ++ treatment. Single RST screening and positive test by Dual Trep/Non-Trep RST and + treatment. All women presenting at ANC receiving treatment.	Pregnant women	1.25% in Peru, 5.14% in Tanzania, and 9.04% in Zambia	True cases treated, missed cases, over-treated, total cost, cost per women screened, cost per women treated, cost per DALY averted, and ICER
Bristow et al. (17)	Malawi	Single HIV rapid test. Dual HIV and syphilis rapid test. Single HIV and syphilis rapid tests. HIV rapid and syphilis tests based on laboratories.	Antenatal patients	(1) 09% in HIV uninfected pregnant women, (2) 17% in HIV infected pregnant women	Adverting adverse birth outcomes, total cost, DALYs lost, CER, and ICER
Hersh et al. (34)	the United States	Single screening at the first prenatal visit. Repeat screening in the third trimester.	Pregnant women	0.00419%	Adverting adverse birth outcomes, total costs, and QALYs
Huntington et al. (35)	The United Kingdom	Universal repeat syphilis screening in late pregnancy. Repeat screening for high-risk women only.	Pregnant women	0.035% at the start of pregnancy, 0.0017% with syphilis during pregnancy	The cost to avoid adverse birth outcomes and the number needing to be screened/treated to avoid adverse birth outcomes.
Romero et al. (36)	Brazil	On-site ICS test and treatment on the same day. Off-site VDRL+TPHA test and treatment at the follow-up visit.	Pregnant women aged between 10 and 49 years	1.2%	Mother-to-child syphilis transmission, adverting adverse birth outcomes, DALYs lost, and ICER
Rodriguez et al. (18)	South Africa, Kenya, Colombia, and Ukraine	HIV and syphilis screening at the first ANC visit. Rapid HIV and syphilis test at the first ANC visit. HIV and syphilis tests at the first ANC visit and repeat screening during late ANC and at delivery. Rapid HIV and syphilis test at the first ANC visit and repeat screening during late ANC and at delivery.	Pregnant women	1.2% in Kenya, 2.0% in South Africa, 0.41% in Colombia, and 2.5% in Ukraine	Total Cost, total DALYs, incremental cost, and ICER

ANC, Antenatal care; CER, Cost-effective ratio; DALY, Disability-adjusted life year; HIV, Human immunodeficiency virus; ICS, Immunochromatographic strip; ICER, Incremental cost-effective ratio; MTCT, Mother-to-child transmission; POC, Point-of-care; RPR, Rapid plasma reagin; RST, Rapid syphilis tests; TPHA, Treponema pallidum hemagglutination assay; and VDRL, Venereal disease research laboratory test.

Table 4 presents information on the following categories: perspective, time horizon, currency and reference year, discount rate, costs included, and main results. In addition to the four unreported studies’ research perspectives, four were social perspectives, eight were health system perspectives, and one was provider perspective. The cost of most studies included testing, counseling, treatment, and labor costs. Of the included studies, 10 had a lifetime horizon, one had a gestation period, one had a 4-year time horizon, and one had a 20-year time horizon. The currency used in most of the studies was the US dollar, and only one study used the United Kingdom pound sterling. Among the included studies, two studies did not report the discount rate, and one study did not need to report the discount rate due to less than 1-year time horizon. The discount rate of 10 studies was 3%, two studies were 5%, and one study was 3.5%. One study used 3% for effect discount and 5% for cost discount.

**Table 4:** The perspective, time horizon, currency and reference year, discount rate, costs included, and main results of the 17 studies.

	Perspective	Time horizon	Currency and reference year	Discount rate	Costs included	Results
Vickerman et al. (25)	Not reported	Not reported	2005 US $	Not reported	Cost for RPR intervention(including start-up, shaker, centrifuge, fridge, training, total drug, RPR test, other supplies, laboratory staff, medical and other staff, and transport for requisitions); cost for POC test intervention (included POC test and penicillin).	In addition to the definitive test, all POC tests saved more DALY than the RPR test on average and averted adverse birth outcomes. Except for Bioline, all POC tests were less cost-effective than RPR tests.
Blandfod 2007 (26)	The perspective of the Eastern Cape Provincial Department of Health	The period of gestation	2002 US $	Not applicable	Total test costs (supplies, labor), supply costs, labor costs, and treatment costs	On-site ICS averted most CS cases, with an incremental cost-effectiveness ratio of $114 per case compared to off-site RPR/TPHA.
Schackman et al. (27)	Societal perspective	A normal life expectancy	2004US $	3%	Labor cost, rapid test cost. RPR test cost, syphilis treatment cost, postnatal care cost, and indirect cost	The CER of rapid testing with immediate treatment was $6.83/DALY and $9.95/DALY in rural settings and urban settings, respectively.
Rydzak and Goldie (28)	Not reported	Lifetime	2004 US $	Not reported	Direct medical costs (including labor, counseling, laboratory equipment, and testing); treatment supplies (including penicillin, swabs, and syringes, and hospitalization for treatment of adverse pregnancy outcomes); and non-medical.	Single ICS screening could save $170,030 per 1,000 women in their lifetime compared to no screening.
Owusu-Edusei et al. (29)	Not reported	Lifetime	2008 US $	3%	Post-test counseling cost, patient cost (including travel, testing time, and treatment time), test cost, treatment cost, and pregnancy outcome cost.	The ICS strategy was the most cost-saving, with a total cost of $76,000 and two adverse pregnancy outcomes per 1,000 pregnancies, which was followed by the Dual-POC strategy with a total cost of $79,000 and 5 adverse pregnancy outcomes.
Kuznik et al. (30)	National health care system	Not reported	2011 US $	3%	ICS test cost, the cost of the nursing time required to administer the test, drug cost, drug delivery equipment cost, and the cost of the nursing time to administer the therapy.	The average cost/DALY averted of syphilis screening was $11 in all 43 sub-Saharan African countries.
Kahn et al. (31)	Societal perspective	4 years	2010 US $	3%	Test cost (the syphilis test kit, labor, and supplies), costs of treatment, and the cost of each mother-to-child transmission of syphilis adverse birth outcomes.	Scaled-up screening and treatment strategy were highly cost-effective, with an ICE of $24 –111 per DALY averted in different scenarios.
Larson et al. (22)	Provider’s perspective	Life expectancy	2012 US $	3%	Test costs, treatment costs, and health workers' training costs.	The cost per DALY saved from screening and treatment strategy was $628. If all positives were treated, the cost per DALY saved falls to $66.
Owusu-Edusei et al. (19)	Chinese national health departments’ perspective	The life expectancy	2010 US $	3%	Test costs, treatment costs, and treatment of adverse pregnancy outcomes costs.	The costs per DALY saved from syphilis-only, HIV-and-syphilis, and HIV-only were $168, $359, and $5636, respectively. The ICER of the HIV and syphilis strategy was $140 per additional DALY avoided compared with the HIV-only strategy.
Albright et al. (32)	Health care perspective	Lifetime	2014 US $	5%	The cost of testing, maternal follow-up and treatment, penicillin desensitization, maternal delivery, and neonatal care.	The incremental cost of universal third-trimester repeat screening was $419,842 per CS prevented.
Kuznik et al. (33)	The perspectives of the national healthcare payer	Not reported	2012 US $	3%	Costs included were the ICS test, three injections of benzathine and penicillin, and nurse wages.	The syphilis screening strategy was with an incremental cost/DALY averted of US$53 in Asia and US$60 in Latin America.
Terris-Prestholt et al. (21)	Not reported	Not reported	2012 US $	3%	The average clinic-level costs for health systems inputs, fixed clinical costs, and variable costs at the patient level to estimate total clinic costs per screening and treatment approach.	Single RST strategy was the most cost-effective, with the cost per DALY averted at $53.69 in Peru, $16.5 in Tanzania, and $16.1 in Zambia.
Bristow et al. (17)	Societal perspective	Life expectancy of the child	2012 US $	3%	Labor costs (counseling, sample collection, preparing and inoculating tests, and reading and recording results), patient costs (travel, testing time cost), test cost, treatment for syphilis, and pregnancy outcome cost.	The dual HIV and syphilis rapid testing strategy was both the least costly ($214.79 per pregnancy) and with the fewest DALYs (108,693) per 100,000 pregnancies.
Hersh et al. (34)	Societal perspective	Life expectancy	2017 US $	3%	Screening test costs, children with CS costs, newborn hospital stay costs, and premature neonates with a variety of outcomes.	The ICER for repeat screening was -$14,098 compared with a single screening cost.
Huntington et al. (35)	The UK healthcare system perspective	Lifetime	2017/2018 UK pound sterling	3.5%	Syphilis screening cost, the management of women diagnosed with syphilis in pregnancy cost, intrauterine fetal demise cost, preterm delivery cost, term delivery cost, neonatal death cost, CS testing and treatment cost, CS neonatal screening cost, CS lifetime healthcare cost, and CS lifetime health and social care cost.	The ICER of the repeat screening strategy was £120,494 for a lifetime.
Romero et al. (36)	The public health system perspective	Lifetime	2015 US $	3% (effects)	Costs of tests, value of personnel time, treatments (mother and child), and inpatient care (CS).	The rapid POC test and immediate treatment strategy were cost-effective with an incremental cost of $298.08 per DALY averted.
				5% (costs)		
Rodriguez et al. (18)	Healthcare system perspective	20 years	2017 US $	5%	HIV costs (included third-generation rapid screening, true-positive screening tests, false-positive screening tests, maternal ART, infant ARV prophylaxis, maternal PrEP, and infant ART).	The strategy of dual rapid HIV and syphilis test at the first ANC visit and retesting during late ANC and at delivery was cost-effective compared with HIV and syphilis tests at the first ANC visit strategy with the ICER of $270 in Kenya, $260 in South Africa, $2207 in Colombia, and $205 in Ukraine.
					Syphilis costs (including RPR test screening, TPHA test screening, dual test screening, Benzathine benzylpenicillin injection, maternal treatment, intravenous benzathine benzyl penicillin, infant treatment, and pediatric inpatient).	

ANC, Antenatal care; ART, Antiretroviral therapy; ARV, Antiretrovirals; CER, Cost-effective ratio; CS, Congenital syphilis; DALY, Disability-adjusted life year; HIV, Human immunodeficiency virus; ICS, Immunochromatographic strip; ICER, Incremental cost-effective ratio; IUFD, Intrauterine fetal demise; POC, Point-of-care; PrEP, Pre-Exposure prophylaxis; RPR, Rapid plasma reagin; RT, A rapid point-of-care test; RST, Rapid syphilis tests; ST, A laboratory-based standard test; TPHA, Treponema pallidum hemagglutination assay; UK, The United Kingdom; US, The United States; and VDRL, Venereal disease research laboratory test.

#### Cost-effectiveness of the different tests

Vickerman et al. (25) estimated the cost-effectiveness of using the rapid POC syphilis tests compared with rapid plasma reagin (PRP) in the Mwanza antenatal syphilis screening. POC tests saved more disability-adjusted life years (DALYs) than the RPR test but were less cost-effective than the RPR test unless the cost of POC was $0.63. Rydzak et al. assessed the cost-effectiveness of three screening strategies, namely, conventional two-step screening using an RPR test followed by a *Treponema pallidum* hemagglutination assay (TPHA) confirmatory test, single-visit rapid RPR, and single-visit rapid immunochromatographic strip (ICS) compared with no screening. They found that compared with no screening, single visits with ICS was a cost-saving strategy of US$170,303 per 1,000 women over their lifetime (28). Owusu-Ediusei et al. compared the health and economic outcomes of four testing/screening algorithms, namely, the dual-POC test, RPR+TPHA algorithm, an onsite PRP testing, and ICS testing, and the results showed that the dual-POC test was the most cost saving (saved $30,000) in resource-poor and high prevalence settings (29).

#### Cost-effectiveness of syphilis tests and treatments

The scaling-up of syphilis screening and treatment strategy was likely to be highly cost-effective in a wide range of settings (31). Larson et al. (22) reported on a model-based study and found that syphilis screening was only cost-effective for reducing adverse birth outcomes if positive patients were treated. ICS testing and subsequent treatment strategy were proved to be highly cost-effective compared with no testing and no treatment in Sub-Saharan Africa and low- and middle-income countries in Asia and Latin America (30–33). The ICS test, which involved the same-day treatment of those testing positive, was cost-effective compared to PRP/TPHA that needed patients returning for results and treatment in high maternal syphilis prevalence settings such as the Republic of South Africa and Brazil (26, 36). Terris-Prestholt et al. (21) assessed the cost-effectiveness of maternal syphilis screening using rapid syphilis tests (RSTs) detecting only treponema pallidum antibodies (single RSTs) or both treponemal and non-treponemal antibodies (dual RSTs). They found that the single RST and treatment should be the best approach unless the price of the dual RST was significantly reduced (21).

#### Cost-effectiveness of repeat syphilis screening

Albright et al. (32) estimated the cost-effectiveness of universal third-trimester syphilis repeat screening in the United States compared to no-repeat screening, and they found that the repeat screening approach was at a high healthcare cost to prevent adverse outcomes. The universal repeat screening was also proved to be not cost-effective in the United Kingdom setting of a low syphilis prevalence (35). However, Hersh et al. (34) reported that the strategy of screening all women during the first and third trimesters was cost-effective and improved maternal and neonatal outcomes in the United States.

#### Cost-effectiveness of integrated HIV and syphilis screening

The integrated screening strategy using a rapid syphilis test as a part of prenatal HIV test would prevent CS cases and stillbirths and would be cost-effective in Haiti (27). In addition, Bristow et al. (17) found that a dual HIV and syphilis test was even cost-saving. Owusu-Edusei et al. assessed the health and economic outcomes of four different strategies of HIV and syphilis screening in pregnant women, namely, no screening, screening for HIV only, screening for syphilis only, and screening for both HIV and syphilis. The results showed that prenatal HIV screening, including syphilis screening, would be substantially more cost-effective than HIV screening alone in China (19). Rodriguez et al. (18) modeled and evaluated the cost-effectiveness of dual maternal HIV and syphilis testing during ANC and retesting during late ANC strategies in high and low HIV prevalence countries, and they found that the dual rapid diagnosis test was cost-saving compared with individual HIV and syphilis tests. The strategy of retesting during late ANC with a dual rapid diagnostic test was evaluated to be cost-effective compared with the strategy of screening syphilis and HIV with the dual rapid diagnosis test in the first ANC visit (18).

## Discussion

Pregnant women infected with syphilis not only endanger their own health but also cause intrauterine infection of the fetus, resulting in abortion, premature birth, stillbirth, or delivery of CS children, which greatly endangers the health of the offspring (37, 38). Based on the prevalence and harm of CS, the WHO put forward a global action plan for eliminating CS in 2007 and formulated corresponding strategies (9). Syphilis screening during pregnancy and treatment of positive pregnant women can effectively reduce MTCT and improve the eugenic rate. Studies have shown that pregnant women with syphilis can effectively prevent adverse pregnancy outcomes through early diagnosis and intervention (3, 38, 39). To select the best screening strategy for syphilis during pregnancy, a detailed and comprehensive economic evaluation is needed to provide a basis for health policymakers. This systematic review aimed to synthesize the results of cost-effectiveness analyses of screening for prevention of MTCT of syphilis and summarize the available evidence.

The cost-effectiveness of universal early pregnancy syphilis screening was evaluated in developed countries, developing countries, and countries with limited resources. The results of the economic evaluation show that in developed countries or countries with limited resources, screening pregnant women for syphilis is cost-effective compared with no screening. In addition, early pregnancy screening in areas with high syphilis incidence is not only cost-effective but there is also economic value in intervening in syphilis screening in countries with very low syphilis prevalence. Screening compliance may affect the cost-effectiveness of screening. In addition, women with poor compliance generally carry a higher burden of syphilis. Focusing on improving the screening rates in this population may improve the health benefits of screening. The treatment rate of positive pregnant women can also affect the cost-effectiveness of screening strategies, mainly because screening can timely detect positive pregnant women and intervene on time to effectively reduce the incidence of CS, thereby improving the quality of life of newborns and reducing economic costs. All of the included studies showed that the maternal syphilis screening strategy was cost-effective. However, the specific policy context and economic development of different countries must be taken into account in the analysis, and the results of other countries cannot be directly used.

Serological tests for syphilis include the treponema pallidum test and the non-treponema pallidum test. The titer of the non-treponema pallidum test is associated with syphilis activity. Most patients with syphilis have passed standard treatment and have turned negative for non-treponema pallidum but remain positive for treponema pallidum or even lifelong positive. At present, a non-treponema pallidum antigen test (such as RPR) is used as the primary screening, and a treponema pallidum antigen test (such as TPHA) is used as the confirmation strategy. However, because the screening for syphilis often needs to be based on laboratory testing, it is largely limited by the existing technical conditions, especially in developing countries and primary care institutions.

In addition, the included literature compared the cost-effectiveness of various testing methods, summarized the results of different testing methods, and further evaluated the best options for syphilis screening. The main reason is that rapid testing can effectively integrate testing and treatment, improve treatment compliance among positive pregnant women, and effectively reduce the occurrence of adverse pregnancy outcomes, especially in regions with limited resources. In 2004, the WHO put forward the urgent need for new POC diagnostic tests for bacterial sexually transmitted infections and formulated standards to highlight that POC testing should be affordable, have reliable sensitivity and specificity, simple to operate, require no training, quick, no need for refrigeration supply chain, and no additional laboratory equipment (40, 41). Most of the available syphilis POC tests detect antibodies against the syphilis pathogen *Treponema pallidum* (TP) and can be used as a screening test. Most studies included showed that POC tests were cost-effective and would improve both maternal and infant outcomes compared to the traditional tests (28, 29). The study in Tanzania found that the high cost of the POC test might limit its cost-effectiveness value (25).

Although repeated screening is recommended for high-risk pregnant women (42–44), the evidence for economic evaluation is still inadequate. The results of the articles included in this study show that syphilis repeat screening in the third trimester of pregnancy might not be cost-effective, but the areas implementing that intervention were restricted to developed countries, such as the United Kingdom and the United States (32, 34, 35). The conclusions remain heterogeneous. Albright et al. (32) found that it was unlikely that the universal repeated screening for syphilis in the third trimester of pregnancy has cost-effectiveness value in the environment with low syphilis prevalence in the United States. In contrast, this study did not use quality-adjusted life years as a measure of health outcomes, which could underestimate the impact of CS on health quality. However, Hersh et al. (34) showed that repeated syphilis screening on third-trimester pregnant women in the United States may have cost-effectiveness value. In Hersh’s study, the incidence of maternal syphilis was 0.00419%, while the probability of syphilis infection during pregnancy was 0.012% (34). The higher incidence of syphilis infection during pregnancy makes repeated screening during pregnancy more cost-effective. However, the optimal interval between the first and second tests is not considered, and it is unclear whether the cost-effectiveness deteriorates when the interval is shortened. Therefore, the optimal time interval needs to be well-evaluated.

Testing coverage for HIV is often higher than for syphilis, and the integration of syphilis and HIV testing may improve the coverage of syphilis testing (15, 45, 46). The genital ulcer disease caused by syphilis infection can increase the risk of horizontal transmission of HIV among the maternal population, making the infection rate of syphilis-infected maternal HIV higher than that in the general maternal population (47–49). Syphilis infection during pregnancy can cause pathological changes in the placenta, destroying the normal function of the maternal-fetal circulatory system, thus affecting the growth and development of the fetus and promoting the MTCT of HIV (50, 51). The combined screening of syphilis and HIV for pregnant women can not only significantly improve the coverage of screening but the results of the overall cost-effectiveness studies included in this study showed that the integrated screening was cost-effective and also cost-saving (17–19, 27).

The evaluation results of most of the studies included are conservative, and the effects of screening interventions on high-risk behaviors in mothers are not considered in the models, which may underestimate the cost-effectiveness of screening. Some studies have shown that the transmission rate of people who do not know their infection is higher than those who know their conditions (52, 53). Thus, maternal screening may reduce the risk of sexual transmission, as well as the potential impact of encouraging positive maternal partners to be tested, but these effects have not been considered. In addition, most studies aimed to assess the health effects on infants and do not include the health effects on mothers after pregnancy, and the health output of maternal syphilis screening may be underestimated. It is also important to assess the negative benefits, such as the psychological burden on the mother, as these may significantly affect the quality of life and thus the ultimate cost-effectiveness of screening. Most studies have been conducted under the following assumptions: (1) syphilis prevalence is the same among women who accept screening and those who refuse, (2) the side effects of antiviral treatment are ignored, (3) the costs do not cover indirect medical costs, and (4) syphilis screening results do not significantly affect pregnant women’s reproductive choices.

The cost-effectiveness analysis studies generally discount future costs and health outcomes. Most of the studies included in this study used discount rates ranging from 3 to 5%. One study used a discount rate of 5% for costs and 3% for health outcomes (36). However, the best discount rates and whether to use different discount rates for costs and health outcomes remain controversial.

Sensitivity analysis allows for a more accurate assessment of the reliability of cost-effectiveness analysis results and minimizes the uncertainty caused by the lack of accurate data on the key parameters. All included studies used sensitivity analysis to evaluate the stability of reported results, including one-dimensional sensitivity analysis and probability sensitivity analysis. These studies mainly focused on the impact of changes in the prevalence, sensitivity, and specificity of syphilis testing, as well as the cost and screening acceptance rate on the cost-effectiveness of screening. However, it is increasingly difficult to accurately predict health outcomes and lifetime costs as screening costs, therapeutic regimens, and treatment costs are constantly changing. There was also heterogeneity in the research perspectives of the included studies, with some studies only considering costs incurred in the healthcare perspective and not including indirect costs due to time costs, which may also have an impact on the results, and others were analyzed from the whole society perspective.

This study also has some limitations. First, the literature included in the study is mainly concentrated in a small number of countries, mainly in Africa, which may lead to an overestimation of the actual infection rate. Second, only three studies have evaluated related issues in developed countries, which may also lead to limitations in extrapolating our findings to developed countries. Third, most of the studies included in this study focus on more specific local situations, potentially creating limitations in generalizability. Finally, this study included only the literature published in English, which may have had a language bias effect on the results, leading to the miss of some other potentially important studies.

## Conclusion

Overall, this study presented a systematic review of published evidence on the cost-effectiveness of syphilis screening in pregnancy to provide a scientific basis for the development of appropriate syphilis screening strategies. The cost-effectiveness of syphilis screening in pregnancy compared with no screening has been widely established. In resource-limited areas with high syphilis incidence, rapid testing has the best cost-effectiveness value. In developing countries with better health resources, screening strategies based on more accurate serological tests are preferable. Repeat syphilis screening strategies in the third trimester are less likely to be cost-effective in developed countries, where the incidence of syphilis is low. While implementing the screening, it is even important to improve compliance with maternal screening and positive maternal treatment rates. Indeed, those with poor adherence were more likely to be at high risk. Furthermore, syphilis screening in combination with HIV screening should be advocated and promoted. To effectively prevent MTCT of syphilis and eliminate CS, in addition to adopting the strategy of syphilis screening and treatment during pregnancy and childbirth, we also need to strengthen education and supervision, pay attention to the shame and fear of syphilis on pregnant women and their families, and improve the compliance of syphilis screening and treatment for pregnant women. In addition, strengthening international cooperation to comprehensively improve and enhance the availability of quality medical care is also the key to achieving the goal of eliminating CS.

## Data availability statement

The original contributions presented in the study are included in the article/supplementary material, further inquiries can be directed to the corresponding author.

## Author contributions

MZ: Conceptualization, Data curation, Formal analysis, Funding acquisition, Writing – original draft, Writing – review & editing. HZ: Data curation, Writing – review & editing. XH: Data curation, Writing – review & editing. HQ: Methodology, Writing – review & editing. JX: Methodology, Writing – review & editing. FX: Writing – review & editing. CS: Writing – review & editing. JH: Writing – review & editing. YC: Writing – review & editing. MH: Conceptualization, Funding acquisition, Writing – review & editing.
